# Budesonide Attains Its Wide Clinical Profile by Alternative Kinetics

**DOI:** 10.3390/ph17040503

**Published:** 2024-04-15

**Authors:** Ralph Brattsand, Olof Selroos

**Affiliations:** 1 Independent Researcher, 68150 Kristinehamn, Sweden; 2 Independent Researcher, 25266 Helsingborg, Sweden; olof.selroos@telia.com

**Keywords:** inhaled corticosteroids, budesonide, cortisol, kinetics, pharmacological profile, clinical profile, asthma, guidelines

## Abstract

The introduction of inhaled corticosteroids (ICSs) changed over a few decades the treatment focus of mild-to-moderate asthma from bronchodilation to reduction in inflammation. This was achieved by inhaling a suitable corticosteroid (CS), giving a high, protracted airway concentration at a low total dose, thereby better combining efficacy and tolerance than oral therapy. Successful trials with the potent, lipophilic “skin” CS beclomethasone dipropionate (BDP) paved the way, suggesting that ICSs require a very low water solubility, prolonging their intraluminal dissolution within airways. The subsequent ICS development, with resulting clinical landmarks, is exemplified here with budesonide (BUD), showing that a similar efficacy/safety relationship is achievable by partly alternative mechanisms. BUD is much less lipophilic, giving it a 100-fold higher water solubility than BDP and later developed ICSs, leading to its more rapid intraluminal dissolution and faster airway and systemic uptake rates. In airway tissue, a BUD fraction is reversibly esterified to intracellular fatty acids, a lipophilic conjugate, which prolongs airway efficacy. Another mechanism is that the rapidly absorbed bulk fraction, via short plasma peaks, adds anti-inflammatory activity at the blood and bone marrow levels. Importantly, these plasma peaks are too short to provoke systemic adverse actions. Controlled clinical trials with BUD changed the use of ICS from a last resort to first-line treatment. Starting ICS treatment immediately after diagnosis (“early intervention”) became a landmark for BUD. An established dose response made BUD suitable for the treatment of patients with all degrees of asthma severity. With the development of the budesonide/formoterol combination inhaler (BUD/FORM), BUD contributed to the widely used BUD/FORM maintenance and reliever therapy (MART). Recent studies demonstrated the value of BUD/FORM as a generally recommended as-needed therapy for asthma (“anti-inflammatory reliever”, AIR). These abovementioned qualities have all influenced international asthma management and treatment guidelines.

## 1. Development of Inhaled Corticosteroids and Pharmacological Profiles

Corticosteroid (CS) inhalation trials started in the 1950s and 1960s, based on the idea of “copying” the successful topical skin therapy by inhaling CS into the inflamed airway mucosa of asthmatics, but these early trials failed (cortisone and prednisolone showed no efficacy, dexamethasone was effective but with maintained systemic adverse actions) [1]. The first positive outcome appeared in the early 1970s with a chlorofluorocarbon-based aerosol formulation of the potent skin CS beclomethasone dipropionate (BDP), which ameliorated asthmatic symptoms at a lower extent of systemic adverse actions than by conventional oral CS treatment [2]. The underlying reasons were poorly known, but it was speculated that the lipophilic BDP, with its very low water solubility, stayed longer on the “open” airway mucosa, enhancing/prolonging local efficacy and retarding its systemic uptake rate and lowering the systemic adverse risks. Other potent topical CSs were tested with similar outcomes and drug developmental work started to find the best inhalation profile. This had to be more empirical than mechanistically based due to the lack of CS-sensitive airway inflammation models, difficulties with inhalation studies in small animals, and very poor kinetic and drug metabolic knowledge. Thus, subsequent ICS development was mainly based on combining suitable physicochemical properties, potency (later glucocorticoid receptor affinity and selectivity), kinetics, and pharmaceutical prospects. This resulted in the development and clinical introduction of budesonide (BUD), fluticasone propionate (FP), fluticasone furoate (FF), mometasone furoate (MF), and ciclesonide, after rising lipophilicity and falling bioavailability of the orally deposited and swallowed ICS fraction. BDP and ciclesonide are lipophilic prodrugs hydrolyzed in airways into active compounds of lower lipophilicity.

The prevalent view is that ICS’s predominant target is the central airway, where a high CS concentration is reached by inhaling a potent, lipophilic CS (or prodrug) with a long dissolution time and strong tissue binding. This leads to prolonged triggering of airway GC-receptors, mediating the aimed local anti-inflammatory activity. Furthermore, the prolonged dissolution reduces the systemic uptake rate, which, according to the general view, would reduce CS’s systemic adverse risks [3,4]. However, there are open issues for such simplification. One is the restricted knowledge of the best systemic uptake rate and distribution of the airways deposited fraction and bioavailable through the bronchial and pulmonary circulations. Even if the resulting plasma level is lower than by oral CS therapy, it can, during its systemic disposition, mediate both anti-inflammatory and adverse CS actions, which are poorly separable according to the general view. While systemic CS therapy of airways/lung diseases aims for broad anti-inflammatory efficacy at the airways/lung, blood, bone marrow, and lymph node levels, the ICS treatment concept presupposes that efficacy is achievable by anti-inflammatory activity at the airway level and that protracted treatment reduces asthma exacerbation risks.“Local“ inflammation also comprises systemic components, delivering required inflammatory cells and mediators and exacerbations may even be initiated at the systemic level. Thus, when asthma patients were grouped by biomarkers into predominantly airway inflammation (raised fractional exhaled nitric oxide) or mainly systemic inflammation (high blood eosinophil counts), the latter group had better ICS responsiveness [5,6].

## 2. Budesonide Having a Partly Different Developmental and Pharmacological Background

The BUD project started in the late 1960s, aiming for a new topical CS. While earlier topical CS projects sought just the highest topical potency, we related that to the resulting systemic risk, functionally evaluated in partly new screening models [7]. Chemical exploration revealed that non-symmetrical CS 16α,17α-acetals provided better selectivity than the earlier developed and clinically introduced symmetrical 16α,17α-acetonides. Optimization ended in the non-halogenated, less lipophilic CS structure BUD, selected in the mid-1970s. Profile comparison with BDP showed that BUD had double the topical potency and halved the systemic risk, suggesting a potential for inhalation use in asthma [7,8]. This, together with the clinical knowledge that systemic risks hamper CS use, much more in asthma therapy than topically used for dermatoses, switched the project’s prime indication to inhalation use.

By its lower lipophilicity, BUD has 100-fold higher water solubility, giving it a much more rapid dissolution and uptake into airway tissue than possible for the more lipophilic ICS [3,9,10]. This gives BUD a better dose-response relationship than is possible for the more lipophilic compounds, facilitating its dose adaptation to a fluctuating disease. Its affinity for the glucocorticoid receptor (GC-R) is the same as for beclomethasone mono-propionate (BDP’s active component), but just half of the FP and one-third of the FF and MF [3,9]. Regarding kinetics, all ICS have high hepatic first-pass clearance, but their varying lipophilicities affect the volume of distribution, giving longer plasma half-lives for the most lipophilic ICS (BUD 2.8, FP 14, FF 17–24 h) [11]. By these partly deviating physicochemical and pharmacological properties, BUD fits poorly into the current ICS profile dogma, suggesting that BUD reaches its high clinical efficacy and safety (see below) by alternative mechanisms. Subsequent mechanistic investigations revealed that BUD’s more swift airway tissue turnover is compensated; a fraction is intracellularly transferred into BUD-21oleate, a very lipophilic inactive conjugate, which then is slowly hydrolyzed back to BUD [12,13,14,15]. As this conjugation requires a high BUD concentration, it happens mainly within the central airways and much less at the systemic level [12,13,14]. By this, the less lipophilic BUD reaches some airway efficacy prolongation, normally demanding twice daily inhalation but even once daily can work in mild asthma [16].

**Figure 1:**
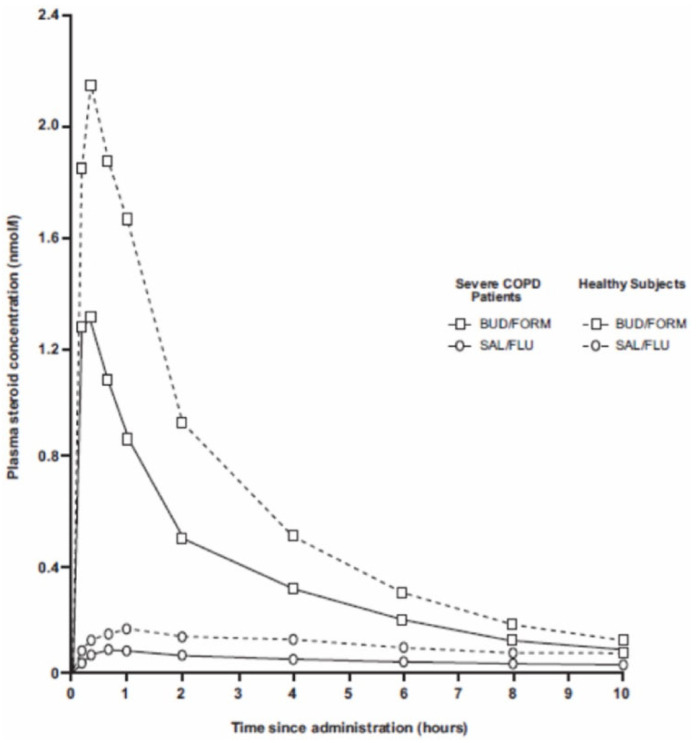
Corticosteroid plasma levels in COPD patients or volunteers, inhaling a single dose of BUD/FORM Turbohaler (400/12 µg) or FP/SALM Diskus (500/50 µg). Reprinted with permission from [17].

But the bulk of airway-deposited BUD is rather rapidly and systemically absorbed; thus BUD’s mean absorption time is 1–2 h, FP’s time is 7 h, and FF’s time is 14 h [3,9,10]. An acute, normal BUD dose inhalation gives a plasma peak at approximately 1 nmol/L, lasting a couple of hours (Figure 1), while a corresponding FP inhalation, due to its slow dissolution and tissue binding, lacks a peak and gives a ten-fold lower, steady plasma concentration [17]. As such, the BUD plasma peak is high and long enough to induce some systemic anti-inflammatory activity, e.g., reduced plasma exudation and depressed lymphocyte activity [9,18]. When asthmatics inhale a normal BUD dose for a week, their bone marrow production of the progenitors of the basophil-eosinophil WBC lineage is decreased [19]. This shows that inhaled BUD also affects the systemic components of ongoing airways/lung inflammation and that, to some extent, mimics mechanisms responsible for systemic CS’s efficacy. The systemic anti-inflammatory potential of the more lipophilic ICSs is less investigated. There is just one study where a high, acute dose of FP or BUD was compared with the outcome that only BUD affected blood WBC numbers and activity [20].

Even if inhaled BUD mediates these systemic anti-inflammatory effects, it does not provoke a similar level of CS’s systemic adverse actions (impaired HPA-axis relationship, metabolic transformations) [21]. We propose that BUD’s pulsatile systemic kinetics enable it to exploit a CS differentiation mechanism programmed for endogenous cortisol to vary its actions by changing the production/release rate [9]. Thus, cortisol’s protection against short-term stress (including injury/inflammation) is favored by high/short release, while its protection against chronic stress (including starvation) is triggered by a more continuous release, which even can be low (e.g., in endogenous Cushing’s disease) [22].

In conclusion, BUD’s rapid systemic uptake of the airway-deposited bulk fraction with a following swift plasma turn-over has two preferences: one, it adds some systemic anti-inflammatory activity; and two, it lowers the systemic adverse risks, contrary to common belief. This, combined with its airway prolongation by reversible esterification, indicates alternative mechanisms for reaching effective and safe inhalation treatment of airway diseases.

## 3. How Budesonide Made Inhaled Corticosteroids First Line Treatment of Asthma

In the early 1980s, budesonide (BUD) was introduced as a pressurized metered dose inhaler (pMDI). BUD was at least as effective as the previously introduced BDP, but it caused less suppression on the HPA axis [8]. In the following decades, BUD became an important tool to document the efficacy and safety of ICS therapy [23,24,25,26].

A new type of clinical study with BUD was initiated after the observation that inflammation was present in bronchial biopsies of patients with mild asthma long before they experienced asthma attacks [27]. Subsequent studies followed, showing that BUD was superior to the earlier first choice—bronchodilators—as a first-line treatment for patients with persistent asthma [28]. Further studies indicated that an early introduction of BUD was superior to a delayed start of treatment [29,30,31]. Since then, BUD and other ICSs have been the first-line treatment for all types of persistent asthma [32].

## 4. Findings Enhancing Budesonide’s Clinical Profile

An important observation was that patients on maintenance treatment with BUD were protected against maximal airway narrowing when exposed to irritants. This was demonstrated by the methacholine challenge tests [33]. The clinical implication of this result was that, even if exposed to high levels of allergens or irritants, patients on regular maintenance doses of BUD did not experience severe airway narrowing. Likewise, studies with allergen challenges showed that regular BUD treatment for a couple of days started to ameliorate the late allergic reaction and after one week, the immediate reaction, too. The full effect on both was seen after 4 weeks of treatment [34]. This had important implications for exercise-induced asthma in children [35].

## 5. Improvements in Formulation and Lung Deposition of Budesonide

**Pressurized metered dose inhaler.** As mentioned earlier, BUD was introduced in 1981 as a chlorofluorocarbon-containing pressurized metered dose inhaler (pMDI). The use of a pMDI requires good hand-mouth coordination and dyscoordination was a common finding among patients [36]. Even with the correct inhalation technique, the lung-deposited fraction of the drug was only approximately 8–10% [37]. When patients used either BDP or BUD pMDIs, the lung deposition and clinical efficacy were very similar [38].

**pMDI plus spacer.** An improvement in BUD therapy was the development of a large-volume spacer attached to the pMDI [39]. In a cross-over study, subjects randomly received three single doses of budesonide on separate days: 0.5 mg given intravenously and 1.0 mg (0.2 mg × 5) by inhalation from a pMDI with a large-volume spacer (Nebuhaler^TM^), with or without concomitant oral charcoal intake to prevent gastrointestinal absorption. Charcoal intake did not significantly affect the systemic availability or deposition of BUD. The systemic availability was 36 ± 14% (metered dose, mean ± SD) with charcoal and 35 ± 10% without. The pulmonary deposition was 36 ± 14% with charcoal and 34 ± 11% without. The use of the spacer reduced or removed the coordination problem and the oropharyngeal deposition of drugs was minimized. At the same time, lung deposition more than doubled compared with a pMDI without a spacer [39]. 

**Multidose dry powder inhaler (Turbuhaler).** A further improvement in BUD delivery was the development of the multi-dose, inspiratory flow-driven dry-powder inhaler Turbuhaler^R^, which also doubled the airway deposition of inhaled drugs compared with the pMDI alone [40]. The lung-deposited dose via the pMDI was 15% at an inspiratory flow of 40 L/min and 32% via Turbuhaler at a flow of 52 L/min. In another study that included BUD Turbuhaler, the lung deposition was 28.5 (range 24–33)% with fast and 17.8 (14–22)% with slow inhalation flow, whereas the deposition after inhalation from a BDP pMDI was 8.9 (6–12)% [41]. In a study comparing lung deposition after inhalation of BUD via Turbuhaler in healthy subjects, the deposition was 28% at an inspiratory flow of 58 L/min and 15% at a flow of 36 L/min [42].

The importance of lung deposition was reflected in studies with BUD Turbuhaler. which offered a superior alternative to BUD pMDIs during the one-year maintenance treatment of asthma [43]. When 200 µg of BUD delivered via Turbuhaler was compared with 400 µg BDP pMDI, the BUD treatment was superior to BDP, indicating a twice as high lung deposition and efficacy of BUD compared with BDP [44].

**Suspension for nebulization.** A low lipophilicity of BUD made the development of a suspension for nebulization possible. The lung deposition and systemic availability of BUD inhalation suspension have been determined in a study in healthy adults using pharmacokinetic evaluation [45]. Three different nebulizers were tested. A total of 2 mg (nominal dose) BUD doses via the Pari Inhalierboy (Inhalierboy; Pari GmbH, Starnberg, Germany), Pari LC Jet Plus (Jet Plus, Pari GmbH), and Maxin MA-2 (MA-2; Clinova Medical AB, Malmö, Sweden) jet nebulizers, 4 mg BUD orally, and 0.5 mg BUD intravenously were administered. Lung deposition and systemic availability of BUD were estimated. There were no differences between nebulizers in lung deposition (14 to 16%) or systemic availability (15 to 17%) relative to the nominal BUD dose. Relative to the actual dose inhaled (dose-to-subject), lung deposition and systemic availability were statistically, significantly higher for the Jet Plus (58 and 63%, respectively) and MA-2 (59 and 64%, respectively) nebulizers than the Inhalierboy (36 and 44%, respectively). The Inhalierboy produced larger aerosol droplets than Jet Plus or MA-2 nebulizers (7-, 5-, and 3-micron mass median diameters, respectively) and delivered a higher dose-to-subject than the other two nebulizers. Relative to the nominal dose, lung deposition and systemic availability of BUD were similar via the three nebulizers tested [45]. When using nebulization, it is therefore important to know the characteristics of the nebulizer used.

The pharmacokinetic parameters of BUD after nebulization and intravenous administration were studied in preschool children with chronic asthma. Plasma concentrations of BUD were measured for three hours after an intravenous infusion of 125 µg BUD. The children then inhaled a nominal dose of 1 mg BUD through the mouthpiece of a Pari LC Jet Plus nebulizer connected to a Pari Master compressor, and the plasma concentrations of BUD were measured for another six hours. The amount of BUD inhaled by the patient (dose-to-subject) was determined by subtracting from the amount of BUD put into the nebulizer, the amount remaining in the nebulizer after nebulization, the amount emitted to the ambient air (filter), and the amount found in the mouth rinsingwater. Approximately 6% of the nominal dose (26% of the dose-to-subject) reached the systemic circulation of young children after inhalation of nebulized budesonide. This is about half the systemic availability found in healthy adults using the same nebulizer [46].

The use of nebulization became particularly useful for the treatment of asthmatic children under the age of 6 years [47].

## 6. Further Clinical Landmarks with Budesonide

**Oral steroid-sparing capacity** was observed in early studies with BUD [48]. Several studies evaluated the capacity of 800 or 1600 µg BUD per day. An example is the study of 159 patients with moderate-to-severe asthma (FEV_1_ 58% predicted normal) using approximately 20 mg of prednisone per day. Patients were randomized to treatment with 800 µg or 1600 µg BUD per day or placebo. The mean reduction in oral corticosteroid dose (OC) dose was 83% and 79%, respectively, in the BUD groups compared with 27% in the placebo group [49].

**Comparison between oral corticosteroids and inhaled budesonide.** The relative potency of BUD and OC was compared in studies by Toogood et al. [50]. Patients controlled on oral prednisone 15 mg plus BDP 800 µg per day were treated with BUD 800 µg, 1600 µg, or 3200 µg and prednisone 15 mg, 30 mg, and 60 mg on alternate days using a double-blind cross-over design. All doses of BUD were significantly better than any OC dose in improving lung function and symptoms [50,51]. Thus, with BUD, it was possible to stop or significantly reduce the doses of OC. Doses of 800 µg per day or above of BUD were more effective than doses up to 60 mg per day of prednisone on alternate days. 

**BUD as first-line treatment.** As mentioned above, the 1980s treatment of asthma was started with the use of bronchodilators. With the bronchoscopy findings that patients with newly detected mild asthma had obvious inflammatory changes in their airways, the question about what drug to use first became evident. Haahtela et al. included 103 patients with newly detected asthma in a 2-year controlled study [28]. Patients were randomized to receive either BUD 600 µg twice daily or a β_2_-agonist bronchodilator, terbutaline 375 µg twice daily. Treatment with BUD significantly improved lung function and reduced bronchial hyperresponsiveness. No changes were noticed in the bronchodilator group. It became clear that early asthma was better treated with an ICS than with a bronchodilator. 

**Early intervention with budesonide.** The 2-year study by Haahtela [28] was continued with a third treatment year. Patients treated with BUD for two years were randomized to treatment with a low dose of BUD or to placebo. Patients treated with terbutaline from the beginning received BUD in an identical way as those patients treated with BUD from the beginning [29]. The interesting and important finding was that a 2-year delay in starting BUD resulted in a significantly poorer response than early treatment. Patients on low-dose BUD remained well controlled, whereas patients on placebo deteriorated during the third year. A follow-up study also demonstrated that the initial superiority shown for BUD compared to bronchodilators was still present 13 years later [52].

When patients were grouped according to the duration of asthma, the greatest improvements in lung function were seen in patients with the shortest duration of disease [30]. Similar results were seen in a study of children with asthma [53]. 

The world-wide 3-year START study, performed in 32 countries, was a double-blind study investigating the impact of early BUD treatment on the outcomes of asthma [54]. The time to the first severe asthma-related event, defined as an event requiring hospitalization, emergency treatment, or death during the study, was the predefined endpoint. A total of 7241 patients were treated with BUD 200–400 µg once daily or a placebo. During a subsequent 2-year follow-up period, all patients received BUD. Treatment with low-dose BUD reduced the risk of a first severe asthma event by 44%.

The importance of early intervention with ICS in asthma was clearly recognized in the 10-year Finnish Asthma Program [55]. Improved asthma control via early use of ICS reduced the use of health care and disability, resulting in major cost savings. Despite a 3-fold increase in asthma patients during the study period in Finland, the total costs decreased by 14%, from EUR 222 million to EUR 191 million. Costs for medication and primary care visits increased, but overall annual costs per patient decreased by 72%, from EUR 2656 to EUR 749. The Finnish Asthma Program resulted in significant cost savings at both societal and patient levels during a 26-year period [56]. The principles of the program have been followed in several other countries with equally good results [57].

**Dose-response** with ICSs has been difficult to demonstrate. However, with BUD studies in both patients with mild asthma [58] and in patients with moderate-to-severe disease [59], dose-response relationships were noticed. However, doubling the dose was not enough to show statistically significant differences between doses in lung function improvements, but quadrupling of the dose did.

**The safety** of BUD, and other ICSs, was questioned after their introduction, as long-term treatment with OCs was associated with a wide range of side effects. It was therefore not surprising that the early use of ICSs, as first-line treatment, was debated. However, it was obvious that the risk of side effects was dramatically reduced with ICSs compared with oral treatments. The risk of growth retardation in children could, nevertheless, not be neglected as studies reported a small but statistically significant effect on children’s final height [60]. This side effect had still to be weighed against the much higher risks with equally effective oral treatment. The effect was mainly seen during the first months of treatment. Thereafter, a catch-up was observed [61,62]. 

The safety of ICSs has been thoroughly reviewed. When used in approved doses, all ICSs appear safe [21,23,24,25,26].

**Budesonide-formoterol combination**. The first long-acting β_2_-agonist (LABA) was introduced in the mid-1990s and recommended for patients with moderate-to-severe asthma that was not well controlled by ICS alone. In the late 1990s, inhaled formoterol (FORM) was added to the treatment repertoire. Inhaled FORM was found to be rapid-acting in addition to its long-lasting bronchodilation [63,64]. The long-acting property was appreciated as an add-on on top of ICS for patients with moderate-to-severe asthma in order to gain control of the disease. The ICS/LABA combination was better in improving asthma control than the individual components alone [65].

Before fixed-dose combinations of ICS and LABA were introduced, there was a discussion about their safety. The FACET study evaluated the combinations of a medium (400 µg twice daily) and a low-dose (100 µg twice daily) BUD together with FORM (9 µg twice daily) or placebo [66]. The exacerbation rate was the primary variable. The addition of FORM to both doses of BUD and increasing the BUD dose four-fold significantly reduced the rate of both severe and mild exacerbations. Low-dose BUD was not optimal to prevent exacerbations.

In patients with mild persistent asthma not well controlled on ICS alone, the addition of FORM 4.5 µg twice daily to BUD 100 µg twice daily resulted in a significantly reduced rate of severe exacerbations compared with a two-fold higher dose of BUD [67]. These results have been confirmed in a large number of clinical studies with ICS/LABA.

There are few direct comparisons of BUD/FORM with LABA in combination with more lipophilic ICSs. In the majority of controlled clinical studies comparing the efficacy and safety of BUD/FORM versus fluticasone propionate/salmeterol (FP), no clinically important differences were seen [9]. In a real-life study comparing BUD/FORM with fluticasone furoate/vilanterol (FF/VI), patients on FF/VI had lower use of a short-acting β_2_-agonist (SABA) and fewer asthma-related exacerbations, which may indicate better asthma control [68]. 

**Budesonide/formoterol maintenance and reliever therapy—MART.** As FORM is both long-acting and rapid-acting, protocols were developed to evaluate the usefulness of BUD/FORM as both maintenance and reliever therapies. In clinical studies where patients used BUD/FORM as maintenance therapy and the same BUD/FORM or a SABA for temporary relief of symptoms, the results showed a clear advantage for the BUD/FORM regimen as an anti-inflammatory reliever, especially in terms of time to first asthma exacerbation [69]. BUD/FORM MART provides flexibility to control the disease when the anti-inflammatory component is also used as needed.

In an open, randomized 4-week study, FF/VI was compared with BUD/FORM MART in 30 patients [70]. Both groups showed significant improvements in FeNO, FEV_1_, and ACQ5 after 2 weeks. At 4 weeks, BUD/FORM MART showed further improvements and significant differences were now seen versus VIL/FF.

**Budesonide/formoterol as an anti-inflammatory reliever.** Some patients with mild asthma do not use regular maintenance therapy but only reliever medication as needed. Also, as mild asthma has an inflammatory component, it was logical to investigate the efficacy and safety of BUD/FORM used as needed instead of an SABA. Data from two large 1-year double-blind studies [71,72] and pooled safety data [73], as well as efficacy and safety in adolescents [74], provided an alternative treatment option for patients not needing regular medication. Using BUD/FORM as needed gave similar protection against severe exacerbations but with a lower total steroid dose. It cannot be excluded that some of the efficacy is due to the systemic component of BUD described above. 

## 7. Budesonide/Formoterol and Guidelines

An important change in asthma management was presented in the Global Initiative for Asthma (GINA) Report 2019 [75]. For safety, GINA no longer recommends treatment with SABA alone. There is strong evidence that SABA-only treatment, although providing short-term relief of asthma symptoms, does not protect patients from severe exacerbations and that regular or frequent use of SABA increases the risk of exacerbations. GINA now recommends that all adults and adolescents with asthma should receive either symptom-driven (in mild asthma) or daily low-dose ICS-containing controlled treatment, i.e., anti-inflammatory medication, to reduce the risk of serious exacerbations. For mild asthma, treatment should be based on an as-needed low-dose ICS-formoterol (BUD/FORM), or if not available, low-dose ICS taken whenever SABA is taken, or regular ICS or ICS/LABA every day, plus as-needed SABA, or maintenance and reliever treatment (MART) with ICS-FORM, with the reliever being low-dose BUD/FORM or BDP/FORM. These recommendations gave BUD a further prominent place on the therapy ladder, as FORM is the only LABA with a rapid-acting onset of action. BUD/FORM can thus be used both as anti-inflammatory maintenance therapy and as anti-inflammatory reliever medication. These changes in guidelines, mainly affecting BUD/FORM, were possible, as both BUD and FORM exhibit dose-response relationships. This makes the use of one inhaler possible over the entire spectrum of disease severities. 

The more lipophilic ICSs, such as fluticasone furoate, mometasone, and ciclesonide, have the advantage of once-daily dosing as the compounds stay for a longer time in the airways compared with BUD [3,4]. Thereby they provide 24 h protection against provoked bronchial hyperresponsiveness (BHR), where BUD requires more frequent inhalations [76,77]. However, induced BHR does not cover all asthma pathology. As the dose and dose frequency of the lipophilic ICSs are more fixed, they fit less well into current guidelines [78]. Furthermore, the LABA component needs to be rapid-acting, and up to now, only inhaled FORM fulfills this property.

## 8. Conclusions

BUD has been available for more than 40 years, is widely used, and now includes many generic formulations and coformulations. From its early stages in pharmacologic and kinetic work, BUD has contributed to the understanding of disease pathogenesis. 

Results of clinical BUD studies have influenced the development of treatment guidelines: from the role of ICSs as first-line treatment, early intervention, protection against allergen- and exercise-induced bronchoconstriction, useful combinations with FORM resulting in both maintenance and maintenance and reliever (MART) strategies, and BUD/FORM as an anti-inflammatory reliever (AIR), used as-needed.

The safety of BUD both in adults and children has been well recognized. This includes, if an effect at all, a clinically nonsignificant effect on the final height of children using BUD.

The economic impact of early use of ICS has been shown in nation-wide asthma programs. Great cost savings have been seen at both individual patient and societal levels.

BUD studies contributed to a better understanding of asthma pathophysiology, especially for the key impact of airway and systemic inflammation on symptom and exacerbation risks.

There is supporting evidence for the hypothesis that BUD’s profile represents an alternative kinetic mode for ICSs to reach a high efficacy/safety ratio. More investigations are needed to confirm the pros and cons of this alternative mode, especially how a more pulsatile glucocorticoid receptor trigger enables a closer adaptation to disease fluctuations in asthma.
